# V-ATPase C Acts as a Receptor for *Bacillus thuringiensis* Cry2Ab and Enhances Cry2Ab Toxicity to *Helicoverpa armigera*

**DOI:** 10.3390/insects15110895

**Published:** 2024-11-15

**Authors:** Pin Li, Yuge Zhao, Ningbo Zhang, Xue Yao, Xianchun Li, Mengfang Du, Jizhen Wei, Shiheng An

**Affiliations:** 1 Henan International Laboratory for Green Pest Control, College of Plant Protection, Henan Agricultural University, Zhengzhou 450046, China; lipin990826@163.com (P.L.); zyg15824990632@163.com (Y.Z.); zhangningbo0109@163.com (N.Z.); yaoxue983@163.com (X.Y.); dumengfang@163.com (M.D.); anshiheng@aliyun.com (S.A.); 2 Department of Entomology and BIO5 Institute, University of Arizona, Tucson, AZ 85721, USA; lxc@email.arizona.edu

**Keywords:** *Bacillus thuringiensis*, *Helicoverpa armigera*, V-ATPase C, Cry2Ab, binding, toxicity

## Abstract

Cry2Ab is a significant alternative *Bacillus thuringiensis* (*Bt*) protein utilized for managing insect resistance to Cry1 toxins and broadening the insecticidal spectrum of crops containing two or more *Bt* genes. Unfortunately, the identified receptors fail to fully elucidate the mechanism of action underlying Cry2Ab. Previous studies have demonstrated the involvement of vacuolar H^+^-ATPase subunits A, B, and E (V-ATPase A, B, and E) in *Bt* insecticidal activities. The present study aims to investigate the contribution of V-ATPase C to the toxicities of Cry2Ab against *Helicoverpa armigera*. The feeding of Cry2Ab in *H. armigera* larvae resulted in a significant decrease in the expression of V-ATPase C. Further investigations confirmed the interaction between V-ATPase C and activated Cry2Ab protein according to Ligand blot and homologous and heterologous competition assays. Expressing endogenous *HaV-ATPase C* in Sf9 cells resulted in an increase in Cry2Ab cytotoxicity, while the knockdown of V-ATPase C by double-stranded RNAs (dsRNA) in midgut cells decreased Cry2Ab cytotoxicity. Importantly, a higher toxicity of the mixture containing Cry2Ab and V-ATPase C against insects was also observed. These findings demonstrate that V-ATPase C acts as a binding receptor for Cry2Ab and is involved in its toxicity to *H. armigera*. Furthermore, the synergy between V-ATPase C protein and Cry2Ab protoxins provides a potential strategy for enhancing Cry2Ab toxicity or managing insect resistance.

## Simple Summary

Currently, identified receptors do not fully elucidate the mechanism of action for Cry2Ab. Here, the results showed that Cry2Ab decreased *HaV-ATPase C* expression, and there was a confirmed interaction between them. The knockdown of *HaV-ATPase C* reduces Cry2Ab cytotoxicity, while its overexpression enhances it. Additionally, enhanced effects against *H. armigera* result from the combination of Cry2Ab and V-ATPase C. These findings demonstrate that V-ATPase C acts as a functional receptor for Cry2Ab in *Helicoverpa armigera*, and the synergistic interaction between them offers a promising strategy to enhance Cry2Ab toxicity.

## 1. Introduction

Insecticidal proteins from *Bacillus thuringiensis* (*Bt*) produced in transgenic crops are used to control some important pests and has become one of the most successful biotechnology products. The genes encoding members of the Cry1A family were initially employed in transgenic crops, followed by the subsequent utilization of two or more distinct *Bt* proteins that exhibit toxicity towards each specific target pest [1,2]. Cry2Ab is a significant alternative *Bt* protein utilized for managing insect resistance to Cry1 toxins and broadening the insecticidal spectrum of crops containing two or more *Bt* genes. In fact, the successful cultivation of *Bt* cotton pyramids, which incorporate the Cry1Ac and Cry2Ab toxins, has been implemented in the United States, Australia, and India to effectively impede the emergence of resistance [3].

Although Cry2Ab (70 kDa) and Cry1Ac (130 kDa) proteins exhibit different molecular weights [4], they both belong to the three-domain (3d) Cry toxin family. The mechanism of action of 3d-Cry toxins involves their ability to form pores in the midgut cells of insects, resulting in insect death [5]. In general, Cry proteins are activated by target insect midgut proteases; subsequently, the activated Cry proteins interact with midgut receptors. Subsequent to this interaction, toxins aggregate to form pre-pore oligomeric structures and ultimately insert into the cell membrane, leading to pore formation and subsequent insect death [5]. Specifically, Cry2Ab has been reported to possess trypsin cleavage sites, and major activated fragments of the Cry2Ab protein with a molecular weight of 50 kDa have been documented [6]. The reported midgut-binding receptors include APN5 in *Plutella xylostella* [7], ABCC1 and V-ATPase E in *Helicoverpa armigera* [8,9], and the most important receptor, ABC transporter gene (ABCA2) [10,11,12,13]. The helices α4-α5 within Domain I have been implicated by Xu et al. [14], as well as the specific residues N151, T152, F157, L183, L185, and I188 have been implicated by Pan et al. [15] as being involved in the oligomerization process of Cry2Ab, which is closely associated with its insecticidal activity and pore-forming ability.

The interaction between *Bt* Cry toxins and functional receptors in the midgut represents a pivotal stage in the toxicological mechanism of *Bt* toxins [16]. However, despite the identification of some candidate receptors, they do not fully elucidate the mechanism underlying Cry2Ab toxicity and certain instances of cross-resistance. For instance, there is an asymmetrical cross-resistance between Cry1Ac and Cry2Ab observed in pink bollworm [17], as well as cross-resistance between Cry1Ac and Cry2Ab in *H. armigera* [18]. Therefore, it is necessary to identify additional Cry2Ab receptors to better understand the mechanism underlying its action and the occurrence of cross-resistance with Cry1Ac. Recently, several genes related to ATP hydrolysis known as vacuolar H^+^-ATPases (V-ATPases) subunit genes have been found to interact with *Bt* toxins. In *Spodoptera exigua*, V-ATPase B was shown to bind to Cry2Aa and contributed to its toxicity [19]. Similarly, V-ATPase E was reported to bind with Cry2Ab and played a role in its toxicity in *H. armigera* [8]. In *P. xylostella*, V-ATPase B was found to interact with Cry1Ac and might be associated with resistance development against this toxin [20]. Bayyareddy et al. demonstrated an interaction between Cry4Ba and V-ATPase in *Aedes aegypti* [21]. Tanaka et al. discovered that there was binding activity between Cry2Aa and V-ATP synthase A in *Bombyx mori* [22]. Similar findings were reported for V-ATPase synthase acting as a binding protein for Cry1Ac in both *Heliothis virescens* [23] and *H. armigera* [24].

In addition, Cry toxins have been documented to disrupt the functions of V-ATPase, including depolarization of the midgut cell apical membrane potential established by V-ATPase [25,26,27], as well as interference with H^+^/K^+^ antiport [28,29]. Importantly, knocking down V-ATPase C in the midgut through RNAi techniques shows its importance in insect pest management as a target [30]. Considering the impact of Cry toxins on V-ATPase function [25,26,27], the binding capacity of Cry toxins and/or the role of multiple V-ATPase subunits in Cry toxin toxicity [8,19,20,24,31,32], as well as the crucial functions of V-ATPase C in insects [30], we propose that V-ATPase subunit *C* may function as a potential receptor for mediating the binding and toxicity of Cry2Ab in *H. armigera*. Furthermore, it is noteworthy that Cry2Ab and Vip toxins are likely to interact with distinct receptors, as evidenced by the absence of significant positive cross-resistance between Vip3 toxins and Cry2Ab [33,34], as well as prior studies suggesting that the receptors for Vip3Aa differ from those for Cry toxins [8,35,36]. Consequently, we opted for Vip3Aa protoxins as a negative control to experimentally validate our hypothesis regarding the binding affinity of HaV-ATPase *C* towards Cry2Ab and its contribution to the toxicity of Cry2Ab.

## 2. Materials and Methods

### 2.1. Insects, Cell Lines and Bt Toxins

The larvae were raised on an artificial diet in a controlled environment with a temperature of 27 °C, a relative humidity of 75%, and a photoperiod of 16:8 h light/dark [8].

The Sf9 cells of *Spodoptera frugiperda* ovary and the midgut (MG) cells of *Helicoverpa zea* were, respectively, cultured in Sf-900TMII SFM (Thermo Fisher, Shanghai, China) and Ex-cell^®^420 culture medium (SAFC Bioscience, Lenexa, KS, USA) at 28 °C [37].

Cry2Ab and Vip3Aa protoxins and activated Cry2Ab toxin were generously supplied by Insect-Resistant Biotechnology Laboratory, Institute of Plant Protection, Chinese Academy of Agricultural Sciences. The activated Cry2Ab toxin and Vip3Aa protoxin were reconstituted in either Sf-900 II SFM or EXCELL 420 insect serum-free medium for cytotoxicity testing, following the methodology outlined in our previous study [8].

### 2.2. Sample Preparation and cDNA Synthesis

To investigate the impact of Cry2Ab on the expression of *HaV-ATPase C* in newly hatched larvae and fifth-instar larvae, sublethal concentrations (resulting in 30% mortality of newly hatched larvae) were chosen [8]. The concentrations of *Bt* toxins, sample preparation, and cDNA synthesis procedures followed the methodology described by Zhao et al. [8]. In brief, the newly hatched larvae of equivalent size were subjected to a treatment with Cry2Ab, or equal amounts of Na_2_CO_3_ (solvent of *Bt* toxins), and subsequently, a sample comprising 35 larvae was collected at intervals of 3, 6, 12, 24, and 36 h, respectively. A sample comprising eight midgut tissues of fifth-instar larvae corresponding to each treatment were collected at different time points as mentioned above. The experimental design involved the execution of three biology replicates for each treatment. Then, the total RNA was extracted using RNAiso Plus (Takara Biomedical Technology (Beijing) Co., Ltd., Beijing, China). The reverse transcription kit HiScript^®^III RT SuperMix for qPCR (+gDNA wiper) (Vazyme, Nanjing, China) was employed for cDNA synthesis, following the provided instructions.

### 2.3. Real-Time Quantitative PCR Analyses

The real-time quantitative PCR (RT-qPCR) reaction system and process, as well as the calculation methods, were performed following the methodology outlined by Zhao et al. [8]. All the relative primers are listed in Table S1. The reference genes used in this study were *EF-1α* (GenBank: U20129.1) and *β*-*actin* (GenBank: HM629442.1) of *H. armigera*.

### 2.4. Construction of HaV-ATPase C Expression Plasmids

The coding sequence (CDS) of the *HaV-ATPase C* gene (GenBank: XM_021342589.1) was amplified from midgut cDNA samples using *V-ATPase C*-CDS-F/R primers (Table S1). Subsequently, PCR products were recovered following the instructions provided by the FastPure^®^ Gel DNA Extraction Mini Kit (Vazyme, Nanjing, China). The full-length CDS of *HaV-ATPase C* was then cloned into the ZT4 vector. Then, the CDS of the *HaV-ATPase C* gene was cloned into the pIEx-RFP vector, while the pIEx-RFP carrier with a His tag was provided by Professor Zhao Xiaofan from Shandong University. The cloning process involved using Bgl II and Sac I enzyme sites for insertion into the pIEx-RFP vector (a scheme of vector construction of HaV-ATPase C -pIEx-RFP plasmid is shown in Figure S1). EcoR I and Xho I enzyme sites were used to insert CDS into the pGEx-6P-1 vector. Subsequently, these plasmids were carried out by the Clon Express II One Step Cloning Kit (Vazyme, Nanjing, China) and transfected to DH5α cells. Recombinant plasmids were then extracted for subsequent use.

### 2.5. Heterologous Protein Production, Purification, and LIGAND Blotting of HaV-ATPase C

The HaV-ATPase C protein was produced using the aforementioned HaV-ATPase C-pGEX-6P-1 expression plasmid in the *E. coli* expression strain Rosetta (DE3). The production and purification of HaV-ATPase *C* were carried out by Hangzhou HuaAn Biotechnology Co., Ltd. (Hangzhou, China). The heterologous protein production and purification process described in Zhao et al. was employed [8]. The Elabscience^®^ Biotin Labeling Kit (purchased from Elirit Biotechnology Co., Ltd., Wuhan, China) was utilized to biotinylate the activated Cry2Ab and Vip3Aa protoxin, and their successful biotinylation was confirmed by Western blot analysis, as reported in [8]. To assess the interaction between activated Cry2Ab (or Vip3Aa protoxin) and expressed HaV-ATPase C protein, 20 μL of HaV-ATPase C protein along with an equivalent amount of BSA (as a negative control) and 5 μL Marker (Thermo Scientific, Waltham, MA, USA) were resolved on an SDS-PAGE gel, transferred onto a PVDF membrane, and subsequently incubated overnight with blocking buffer (5% BSA in PBST solution). Subsequently, biotinylated activated Cry2Ab toxin and biotinylated Vip3Aa protoxin were introduced into the blocking buffer at a final concentration of 5 μg/mL and incubated for 4 h, respectively. The PVDF membrane was subjected to five consecutive washes with PBST for 5 min each time, followed by incubation with HRP-Streptavidin antibody (diluted 1:10,000 in 5% BSA, purchased from Abbkine, Wuhan, China) for 1 h. After another round of washing with PBST five times for 5 min each, the membrane was subsequently exposed on the Tanon-4600 photographic system.

### 2.6. Saturation and Competition Binding Assays

The saturation and competition experiments were conducted using the aforementioned Ligand blotting assay with some modifications. For the saturation binding experiments, 5 µg of HaV-ATPase C protein was immobilized onto a PVDF membrane and incubated with 5 mL of the blocking buffer (5% BSA in PBST solution) overnight. Then, 10, 20, 30, or 40 µg biotinylated Cry2Ab, for a final concentration of 2, 4, 6, and 8 µg/mL, were added to the blocking buffer and incubated for 4 h, respectively. Lastly, this was incubated with 1:10,000 diluted HRP-Streptavidin antibody (Abbkine, Wuhan, China) and 5% BSA for 1 h, washed 5 times for 5 min each with PBST, and then the membrane was subsequently exposed on the Tanon-4600 photographic system. The saturation experiment was conducted in triplicate, and upon reaching a mass of 30 µg, the binding of biotinylated Cry2Ab to HaV-ATPase C exhibited saturation. For the competition assays, 4 pieces excised from each PVDF membrane blot (3 replicates) were separately incubated with 5 mL of the blocking buffer (5% BSA in PBST solution) overnight. Then, they were incubated with 30 µg of biotinylated Cry2Ab toxin protein and 150 µg (5-fold) of unlabeled Cry2Ab toxin protein or unlabeled Vip3Aa toxin protein in 5 mL of the blocking buffer (5% BSA in PBST solution) and incubated for 4 h, respectively. Later, the results were detected as part of the methods of the above saturation experiment. The saturation and competition experimental process described in Zhao et al. was employed [8].

### 2.7. Production of dsRNAs

The T7 RNAi Transcription Kit (Vazyme, Nanjing, China) was employed for the synthesis of double-stranded RNA (dsRNA) targeting *EGFP* and *HaV-ATPase C.* The protocol for generating 586 bp *EGFP* and 423 bp *HaV-ATPase C* dsRNA has been previously described in Zhao et al. [8].

### 2.8. Cell Toxicity Assays

Relevant studies have been referenced for the execution of cell transfection experiments [8,34]. The Sf9 cell line was employed for transfection with *HaV-ATPase C* -pIEx-RFP or the empty pIEx-RFP vector control, aiming to investigate the gain functions of *HaV-ATPase C*. Conversely, MG cells were transfected with *HaV-ATPase C* dsRNA or the *EGFP* control dsRNA to explore the loss functional of *HaV-ATPase C*. The transfection agent used in these experiments was Cellfectin (Promega (Beijing) Biotech Co., Ltd.). After 24 h of transfection, Sf9 cells transfected with the two plasmids (pIEx-RFP or *HaV-ATPase C*-pIEx-RFP) were observed and imaged using a fluorescent microscope (Leica, Wetzlar, Germany) to confirm successful transfection (resulting in red fluorescence). After 48 h, the transfected cells were reseeded in 96-well cell culture plates (10,000 cells) and incubated for approximately 2 h to allow cell attachment. Subsequently, the cytotoxicity of the treated cells was assessed using Cry2Ab and Vip3Aa toxins at concentrations of 200 μg/mL for expressing *HaV-ATPase C* and 150 μg/mL for knockdown of *HaV-ATPase C* over a duration of 2 h. Finally, cell mortalities were determined under an inverted microscope (Leica, Wetzlar, Germany), following the calculation method described by Zhao et al. [8]. The remaining treated cells from each independent transfection were collected using previously established protocols for subsequent RNA or protein analysis via Western blot with a His antibody to confirm successful transfection efficiency [8,38].

### 2.9. Larvae Bioassays

The susceptibilities of *H. armigera* neonates to PBS buffer (the buffer used to dissolve HaV-ATPase C, PBS), Na_2_CO_3_ (used to dilute *Bt*), Na_2_CO_3_ + PBS (buffer control), Cry2Ab, the mixtures of Cry2Ab and V-ATPase C proteins, Vip3Aa, the mixtures of Vip3Aa and V-ATPase C proteins were conducted by diet overlay bioassays using artificial diet. In the mixtures’ treatments, 0.03 μg/cm^2^ Cry2Ab (or 0.03 μg/cm^2^ Vip3Aa) was mixed with 0.03 μg/cm^2^ (1×), 0.15 μg/cm^2^ (5×), 0.45 μg/cm^2^ (15×) or 1.5 μg/cm^2^ (50×) V-ATPase C, respectively. Each treatment used 72 larvae (divided into three repetitions). The implementation of bioassays has been previously described in Zhao et al. [8].

### 2.10. Statistical Analysis

The significant differences in relative expression levels of *V-ATPase C*, cell mortalities, larval weights, and larval mortalities under the different treatments were compared using the LSD test (DPSSOFT: DPS9.01). The binding abilities of HaV-ATPase C with Cry2Ab were compared for significant differences using Student’s *t*-test. (DPSSOFT: DPS9.01).

## 3. Results

### 3.1. The Expression Level of HaV-ATPase C Following Treatment with Cry2Ab

**Figure 1:**
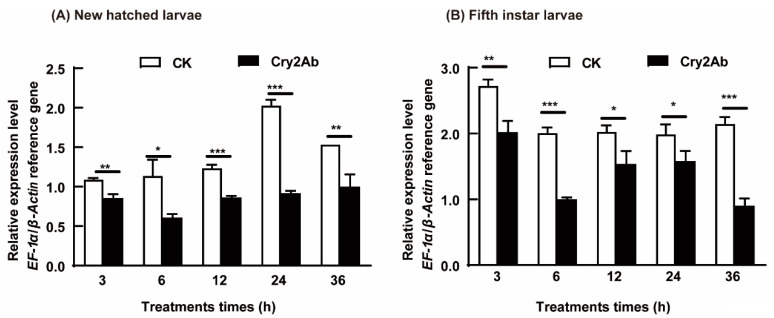
*HaV-ATPase C* expression in newly hatched (**A**) and fifth-instar (**B**) larvae following sublethal Cry2Ab exposure. The standard error of the average from three biological replicates is represented by each error bar. Bars represent the mean (±SD) of three biological replicates. Significant differences at each time point are denoted with asterisks based on Student’s *t*-test (* *p* < 0.05, ** *p* < 0.01, *** *p* < 0.001, DPSSOFT: DPS9.01).

Through RT-qPCR, we observed the ubiquitous expression of the HaV-ATPase *C* gene across all instars, with significantly higher levels detected in the fifth-instar midgut (*p* = 0.0001) (Figure S2). Compared to the neonates fed on the Na_2_CO_3_ diet, those fed on the Cry2Ab protoxin exhibited significantly decreased expression of *HaV-ATPase C* at 3 h (*p* = 0.0018), 6 h (*p* = 0.0117), 12 h (*p* = 0.0002), 24 h (*p* = 0.0001), and 36 h (*p* = 0.0040) (Figure 1A). *HaV-ATPase C* expression level decreased from 2.02 to 0.92, reaching the most significant decrease in the Cry2Ab-treated larvae was observed at 24 h (Figure 1A). Similarly, Cry2Ab treatment significantly suppressed the expression of *HaV-ATPase C* in the fifth-instar larvae at 3 h (*p* = 0.0034), 6 h (*p* = 0.0001), 12 h (*p* = 0.0178), 24 h (*p* = 0.0324), and 36 h (*p* = 0.0001). *HaV-ATPase C* expression level decreased from 2.15 to 0.90 at 36 h, reaching the most pronounced decrease (Figure 1B).

### 3.2. The Special Binding of HaV-ATPase C with Activated Cry2Ab

**Figure 2:**
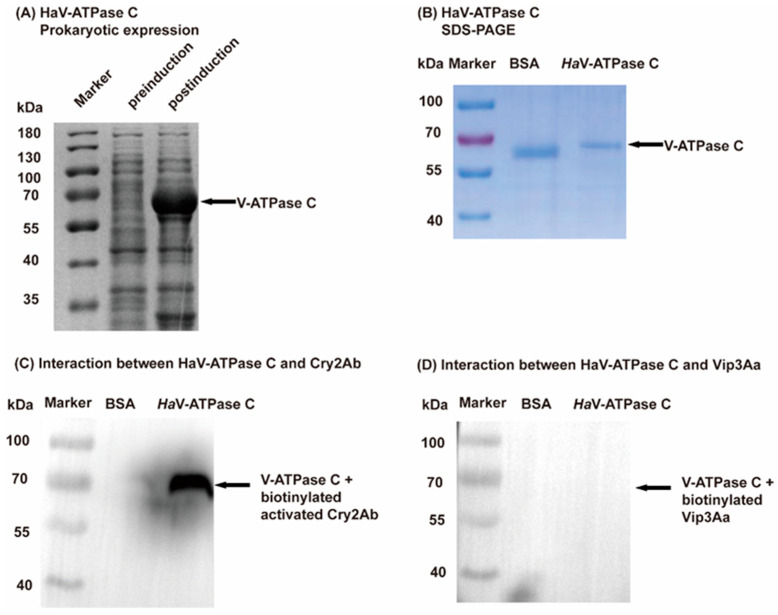
SDS-PAGE analysis of the HaV-ATPase *C* protein and Ligand blotting analysis of the interaction between HaV-ATPase C and *Bt* toxin. (**A**) The prokaryotic expression of HaV-ATPase *C* protein was confirmed. (**B**) SDS-PAGE analysis of the purified HaV-ATPase *C* protein. (**C**) The interaction between HaV-ATPase C protein and activated Cry2Ab toxin. (**D**) The interaction between HaV-ATPase *C* protein and Vip3Aa protoxin.

**Figure 3:**
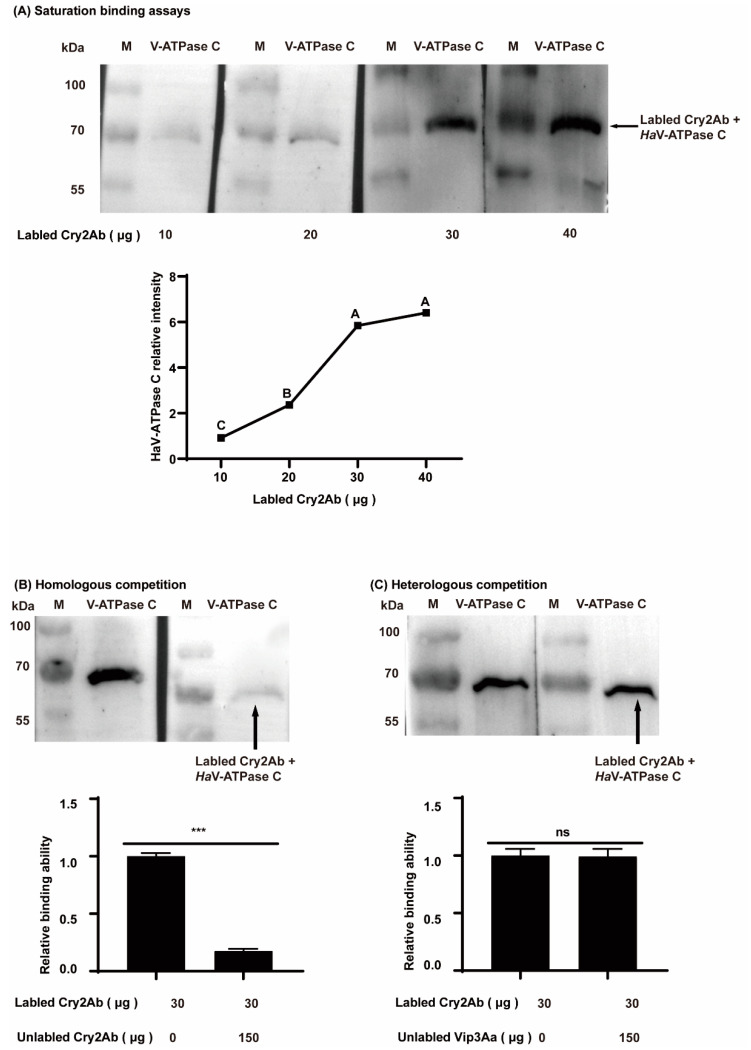
Special binding of HaV-ATPase *C* to activated Cry2Ab. (**A**) Saturation binding assays between HaV-ATPase *C* and activated Cry2Ab. The quantitative determination of the relative intensity of three replicates was conducted using Image J 1.8.0, followed by normalization with the result under the treatment of 30 μg biotinylated Cry2Ab. Bars labeled with different capital letters indicate significant differences (*p* < 0.01) based the LSD test conducted using DPSSOFT: DPS9.01. (**B**) Homologous competition between labeled Cry2Ab with unlabeled Cry2Ab to HaV-ATPase C protein. (**C**) Heterologous competition between labeled Cry2Ab with unlabeled Vip3Aa protoxin to HaV-ATPase C protein. Bars represent the mean (±SD) of three biological replicates. The bars with *** (*p* < 0.001) exhibit significant differences and ns shows no significant differences, based on Student’s *t*-test (DPSSOFT: DPS9.01).

The protein production of HaV-ATPase *C* was confirmed by SDS-PAGE gels (Figure 2A), and the purified HaV-ATPase C protein exhibited the expected molecular weight of 70 kDa (Figure 2B). The Ligand blot analysis revealed that HaV-ATPase *C* protein exhibited binding affinity towards Cry2Ab (Figure 2C), while no interaction was observed with Vip3Aa (Figure 2D). At a fixed load of 5 µg HaV-ATPase C, the binding curve plateaued at approximately 30 µg biotinylated activated Cry2Ab (Figure 3A). Importantly, the HaV-ATPase C specific interaction with labeled Cry2Ab protein can be completed by unlabeled Cry2Ab but not by Vip3Aa (Figure 3B,C).

### 3.3. Overexpressing HaV-ATPase C Increased Sf9 Susceptibility to Activated Cry2Ab

**Figure 4:**
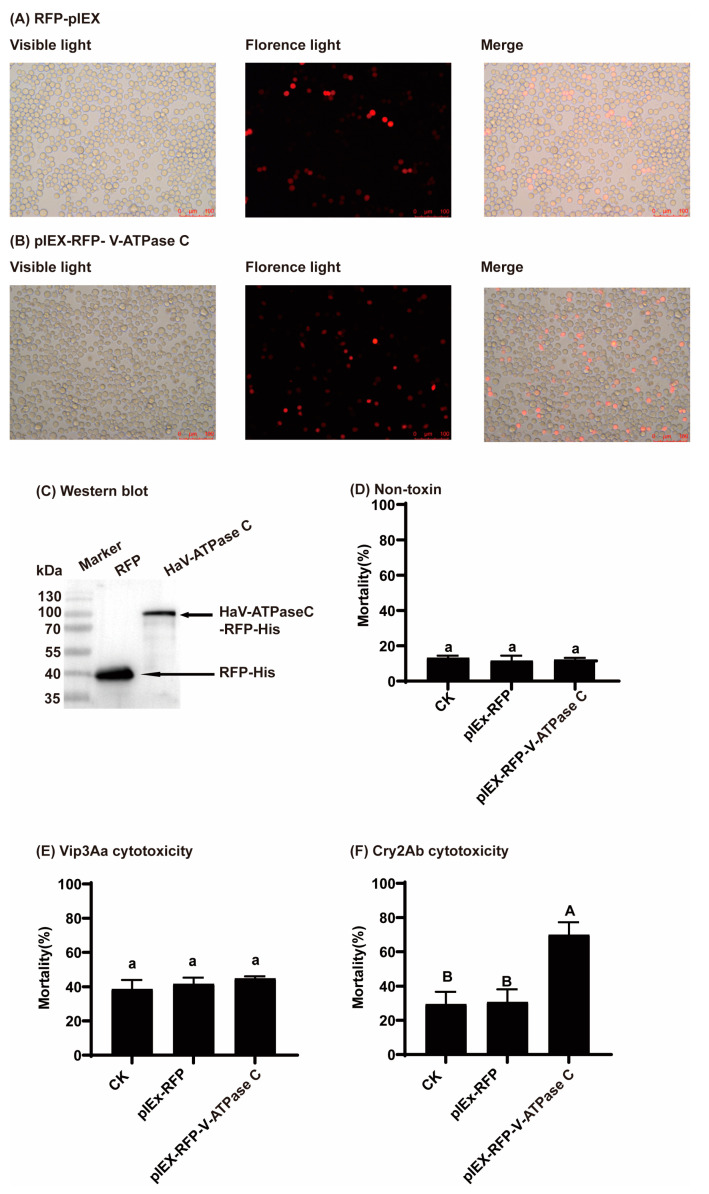
Impact of expression of *HaV-ATPase C* in Sf9 cells on the cytotoxicity exerted by Cry2Ab and Vip3Aa. (**A**) Sf9 cells were transfected with RFP-pIEx plasmid (empty vector, control) 24 h. (**B**) Sf9 cells were transfected with *HaV-ATPase C* -pIEx-RFP plasmid 24 h. (**C**) Expression of HaV-ATPase *C* protein in Sf9 was analyzed by Western blot. (**D**) The cell mortalities of transfected cells in toxin-free treatment. (**E**) Cell mortalities were observed upon exposure to 200 μg/mL Vip3Aa protoxin. (**F**) Cell mortalities were observed upon exposure to 200 μg/mL activated Cry2Ab. Bars represent the mean (±SD) of three biological replicates. The bars with different capital letters (*p* < 0.01) or lowercase letters (*p* < 0.05) exhibit significant differences, as determined by the least significant difference (LSD) test using DPSSOFT: DPS9.01.

The successful expression of *HaV-ATPase C*-pIEx-RFP in Sf9 cells was confirmed through the detection of red fluorescence and Western blot analysis (Figure 4A–C). No significant difference in cell mortality (<15%) was observed among the transfected cells without the toxin treatment (Figure 4D). Similarly, there were no notable differences in the cell mortalities when treated with 200 μg/mL Vip3Aa protoxin (approximately 40%) (Figure 4E). However, the treatment with activated Cry2Ab at a concentration of 200 μg/mL enormously increased the cell mortality by 39.20% compared to that of the cells transfected with the pIEx-RFP recombinant plasmid, indicating that the overexpression of *HaV-ATPase C* enhanced the susceptibility to activated Cry2Ab (*p* = 0.0001; Figure 4F).

### 3.4. Knocking Down HzV-ATPase C Decreased MG Susceptibility to Activated Cry2Ab

**Figure 5:**
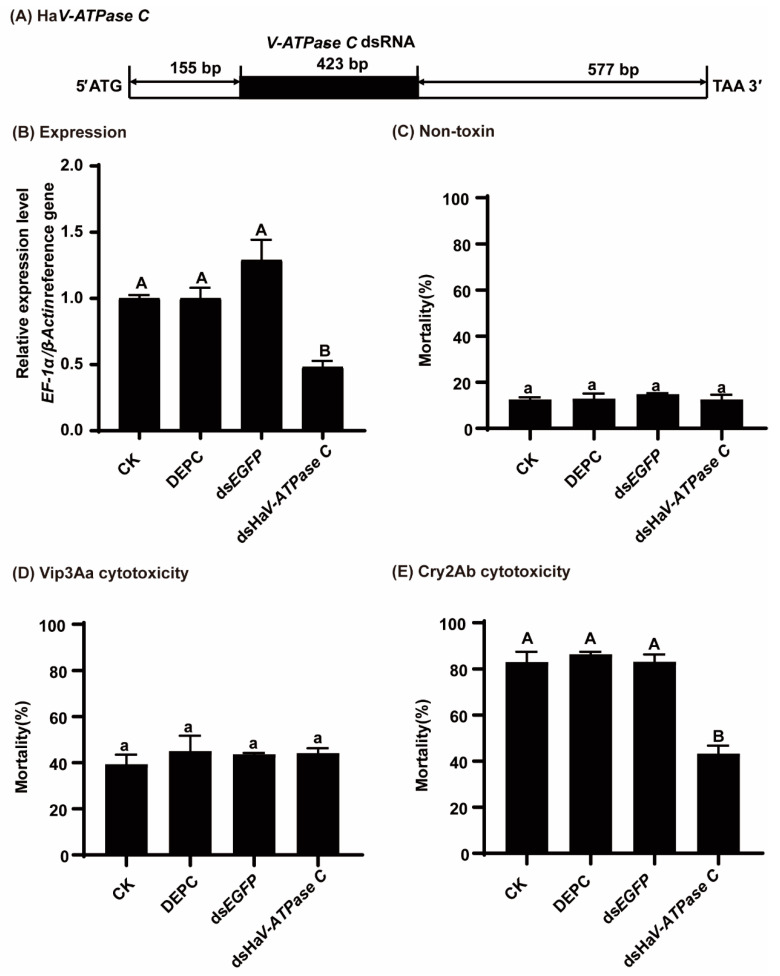
Effects of *HzV-ATPase C* silencing on the toxicity of activated Cry2Ab and Vip3Aa towards MG cells. (**A**) The positioning of the dsRNA fragment within the *HaV-ATPase C* gene. (**B**) The expression levels of *HzV-ATPase C* under different treatments. (**C**) The cell mortalities of different treatments without toxins treated. (**D**) Cell mortalities were observed upon exposure to 150 μg/mL Vip3Aa protoxin. (**E**) Cell mortalities were observed upon exposure to 150 μg/mL activated Cry2Ab. Bars represent the mean (±SD) of three biological replicates. The bars with different capital letters (*p* < 0.01) or lowercase letters (*p* < 0.05) exhibit significant differences, as determined by the least significant difference (LSD) test using DPSSOFT: DPS9.01.

In line with our inference based on the high similarity between *HaV-ATPase C* and *HzV-ATPase C* (99.22%), the RT-qPCR analysis demonstrated significant and effective suppression of *HzV-ATPase C* expression in *H. zea* MG cells upon treatment with *HaV-ATPase C* dsRNA (*p* = 0.0001; Figure 5A,B). The mortalities of the transfected cells were not significantly different without the toxin treatment (<15%) (Figure 5C). Similarly, there was no significant difference in the mortalities with Vip3Aa protoxin treatment (approximately 40%) (Figure 5D). However, knockdown of *HzV-ATPase C* markedly reduced the susceptibility of the MG cells to activated Cry2Ab (Figure 5E). Specifically, the cell mortality induced by 150 μg/mL of activated Cry2Ab decreased by 39.88% compared to *EGFP* dsRNA, 43.15% compared to DEPC water, or 39.74 compared to control medium, respectively (*p* =0.0001; Figure 5E).

### 3.5. The HaV-ATPase C Protein Enhanced the Toxicity of Cry2Ab Protoxin Against Cotton Bollworm

**Figure 6:**
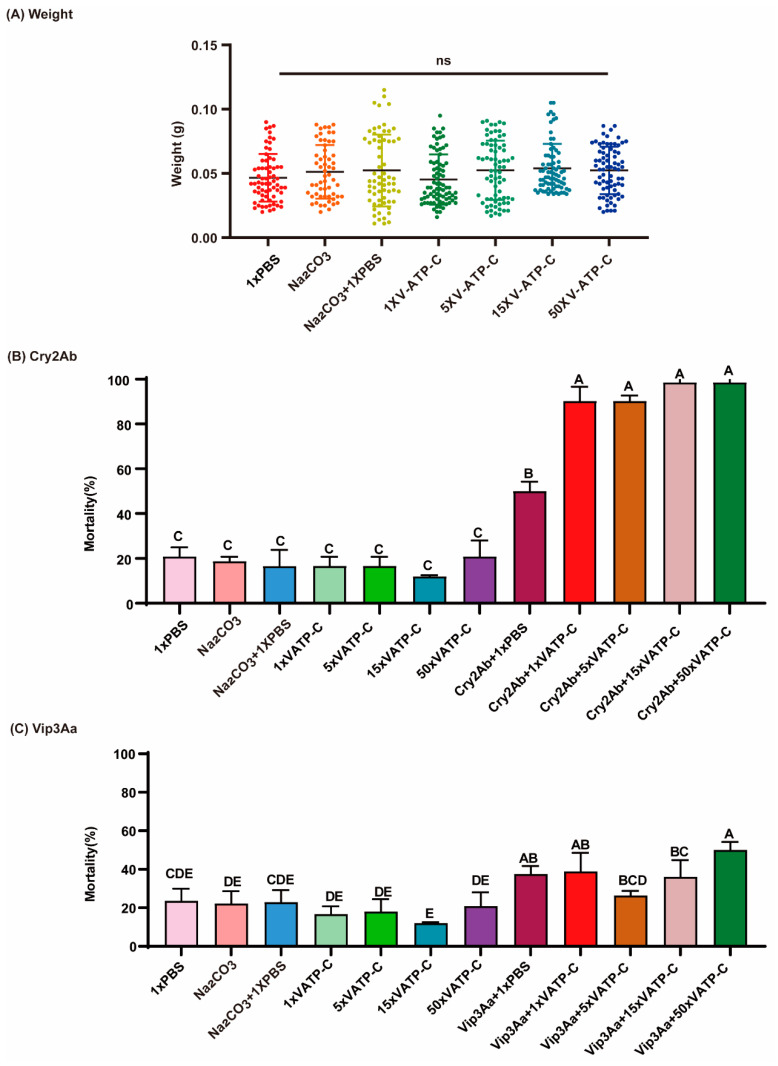
Effect of HaV-ATPase *C* protein on the larvae weight (**A**): the toxicity of Cry2Ab (**B**) and Vip3Aa (**C**). (**A**) The effects of HaV-ATPase C protein on larval weight after 7 days of exposure. (**B**) The impact of incorporating HaV-ATPase *C* on the toxicity of Cry2Ab towards larvae. (**C**) The impact of incorporating HaV-ATPase *C* on the toxicity of Vip3Aa towards larvae. HaV-ATPase *C* dissolved in PBS, and *Bt* toxins dissolved in Na_2_CO_3_. The values 1×, 5×, 15×, and 50× represent the concentrations of V-ATPase C at 1 time, 5 times, 15 times, and 50 times that of *Bt* toxins. The mortality values were expressed as mean ± SD. The ns indicated no significant difference among the effects of HaV-ATPase C protein on larval weight after 7 days exposure. Bars represent the mean (±SD) of three biological replicates. Bars labeled with different capital letters indicate significant differences (*p* < 0.01) based the LSD test conducted using DPSSOFT: DPS9.01.

*H. armigera* neonates were fed with Cry2Ab protoxin or Vip3Aa protoxin alone, or in combination with purified HaV-ATPase C protein at four different ratios (Vip3Aa protein or Cry2Ab protein/HaV-ATPase *C* protein = 1:1, 1:5, 1:15, or 1:50) for a duration of 7 days. The results demonstrated that the administration of purified HaV-ATPase C protein alone did not yield any significant effects on the larval mortality rates and weight (*p* = 0.4769; Figure 6A) (Figure 6A–C). However, the addition of varying amounts of purified HaV-ATPase C protein significantly enhanced the corrected mortality of Cry2Ab but not that of Vip3Aa (this increased the mortalities by40.28% at a ratio of 1:1 and at a ratio 1:5, and increased the mortalities by 48.61% at a ratio of 1:15 and at a ratio of 1:50; *p* = 0.0001; Figure 6B,C), suggesting that the HaV-ATPase C protein enhanced the toxicity of Cry2Ab protoxin against cotton bollworm.

## 4. Discussion

The expression of HaV-ATPase *C* was found to be higher in the first- and fifth-instar larvae (Figure S2), while sublethal exposure to Cry2Ab resulted in a decrease in the expression level of HaV-ATPase C in larvae of both instars (Figure 1). This finding is consistent with previously reported expression patterns of *Bt* receptors after being exposed to *Bt* toxins, such as ALP, ABCC2, ATP synthase subunit α, V-ATPase E, and so on [8,32,38,39,40,41,42,43,44], suggesting the potential involvement of HaV-ATPase C in Cry2Ab toxicity. The expression patterns of ATP-related genes consistently exhibited differential responses following induction with *Bt* toxins. Specifically, certain genes demonstrated decreased expression levels in both *Bt*-resistant strains and *Bt*-fed larvae [32,37,45], whereas others displayed upregulation exclusively in Cry1Ac-resistant strains [46,47]. Remarkably, the downregulated genes positively correlated with the Cry toxicity [8,19,32,38], while the upregulated genes may have exhibited a negative correlation with Cry toxicity [31,46], thereby implying their distinct roles in defense against toxic effects. The downregulated V-ATPase genes may function as receptors for Bt toxins, and the reduction in receptor expression could potentially represent an adaptive strategy to mitigate toxicity [8,19,32,38]. Conversely, the upregulated expression may indicate an enhanced energy-driven defense mechanism in the intestinal tissue, thereby facilitating resistance [38,47], as energy plays a pivotal role in eliciting defense mechanisms against adverse conditions. Insects exhibit precise regulation of the expression of diverse ATPase genes to maintain an equilibrium between energy supply and tolerance against Bt toxins. Investigating the binding properties and functional roles of these ATP-related genes in Bt toxin toxicity could yield valuable insights into the mechanisms underlying insect energy homeostasis and resistance against Bt toxins.

Previous studies have documented the interaction between V-ATPase subunits and Cry toxins in various insect species, including *S. exigua* [19], *P. xylostella* [20], *A. aegypti* [21], *Anthonomus grandis* [48], and *H. armigera* [8,32]. Moreover, HaV-ATP C along with HaV-ATP E and HaV-ATP G constitute the three peripheral stalks of V-ATPases, which are all subunit proteins [49]. Our earlier findings indicated that V-ATPase E bound to Cry2Ab and contributed to its toxicity in *H. armigera* [8]. Consistent with these predictions, a Ligand blot analysis and homologous and heterologous competition experiments demonstrated the interaction between HaV-ATP C and Cry2Ab toxin, but not Vip3Aa (Figure 2 and Figure 3). Functionally, the in vitro gain- and loss-of-function analyses of *HaV-ATPase C* provided valuable insights into its role in Cry2Ab cytotoxicity, but not in Vip3Aa toxicity (Figure 4 and Figure 5). These findings suggested that HaV-ATPase C may act as a specific Cry2Ab receptor. While the exact mechanisms of action remain unclear, HaV-ATPase C, an essential subunit of V-ATPase, may exhibit characteristics similar to those of ABC transporters, such as ABCA2, which acts as a receptor for Cry2Ab. Both types of proteins utilize ATP-driven proton pumps to facilitate the active transport of substrates to the cell membrane. Additionally, HaV-ATPase C may affect the concentration of toxins around V-ATPase through interactions with Cry2Ab, as our findings suggest that HaV-ATPase C is capable of binding to Cry2Ab. This interaction could potentially strengthen the binding affinity between the toxin and V-ATPase, resulting in disruptions to H^+^/K^+^ transport and/or inhibition of (Na^+^, K^+^)-ATPase activity [28,29,50], ultimately leading to insect mortality. However, further comprehensive functional investigations are warranted to fully elucidate the role of HaV-ATPase C in the mode of action exerted by Cry2Ab.

Furthermore, HaV-ATPase C synergistically potentiated the toxicity of Cry2Ab protoxins against *H. armigera* larvae, as demonstrated in this study (Figure 6). This finding is in accordance with previous reports indicating that ALP fragments, ATPs-α fragments, HaV-ATPase E, toxin-binding polycalin fragments, and cadherin fragments enhance the efficacy of Cry toxins against diverse insect species [8,38,51,52,53]. However, the precise mechanisms remain elusive. One plausible explanation could be that the abundance of Cry2Ab molecules (dose) exceeds the available in vivo target molecules on the midgut membrane, leading to the molecules remaining unbound. Upon the addition of a complementary receptor, binding could occur, leading to an increase in larval mortality. Alternatively, the presence of additional HaV-ATPase C mentioned above could affect toxin aggregation, facilitating the interactions between toxins and V-ATPase as well as the subsequent disruption of H^+^/K^+^ transport and/or inhibition of (Na^+^, K^+^)-ATPase [28,29,50], ultimately leading to insect mortality. More research is warranted to unravel the underlying synergistic mechanisms.

In this study, we also ruled out HaV-ATPase C as a possible receptor for Vip3Aa, consistent with our previous findings from our earlier study [8]. Additionally, HaV-ATPase E is also not a receptor for Vip3Aa, suggesting that V-ATPase may not be the target for Vip3Aa [8]. This conclusion is supported by the findings of the nonsignificant resistance interactions between Cry proteins and Vip3Aa [33,34], which indicates that they likely engage with distinct targets. Furthermore, recent studies have identified that Scavenger receptor-C [35] and a chitin synthase protein [36] may be receptors for Vip3Aa, revealing a significant divergence from typical Bt receptors. In summary, this study presents the novel finding of HaV-ATP C’s interaction with Cry2Ab toxins and its role in Cry2Ab toxicity towards *H. armigera*. Furthermore, the synergy of HaV-ATPase C fragment protein and Cry2Ab protoxin offer a promising avenue for enhancing Cry2Ab toxicity or managing insect resistance.

## Data Availability

All the data are in the manuscript and Supplemental Document.

## Supplementary Materials

The following supporting information can be downloaded at: https://www.mdpi.com/article/10.3390/insects15110895/s1, Figure S1: A scheme of vector construction of *HaV-ATPase C* -pIEx-RFP plasmid; Figure S2: *HaV-ATPase C* higher expressed in first and fifth instar larvae; Figure S3: Impact of expression of *HaV-ATPase C* in Sf9 cells on the cytotoxicity exerted by Cry2Ab and Vip3Aa; Figure S4: Effects of *HzV-ATPase C* silencing on the toxicity of activated Cry2Ab and Vip3Aa towards MG cells; Table S1: The primer sequence designed in this experiment.
