# Supplementary material for: V-ATPase C Acts as a Receptor for Bacillus thuringiensis Cry2Ab and Enhances Cry2Ab Toxicity to Helicoverpa armigera
Source: Insects. 2024 Nov 15;15(11):895. doi: 10.3390/insects15110895 (PMC11594778; doi:10.3390/insects15110895)
Supplement: Supplementary file 1 [file insects-15-00895-s001.zip › insects-3235755-supplementary.pdf]

Supplementary Materials

V-ATPase C acts as a receptor for *Bacillus thuringiensis* Cry2Ab and enhances Cry2Ab toxicity to *Helicoverpa armigera*

Pin Li[1#], Yuge Zhao[1#], Ningbo Zhang[1], Xue Yao[1], Xianchun Li[2], Mengfang Du[1], Jizhen Wei[1]*, Shiheng An[1]

[1]Henan International Laboratory for Green Pest Control/College of Plant Protection, Henan Agricultural University, Zhengzhou, 450046, China. lipin990826@163.com (P.L.); zyg15824990632@163.com(Y.Z.); zhangningbo0109@163.com(N.Z.); yaoxue983@163.com (X.Y.); dumengfang@163.com (M.D.); anshiheng@aliyun.com (S.A.)

[2]Department of Entomology and BIO5 Institute, University of Arizona, Tucson, AZ 85721, USA. lxc@email.arizona.edu

*Correspondence: to whom correspondence should be addressed; Jizhen Wei, weijizhen1986@163.com

[#]These authors contributed equally to this work.

Table S1 List of primers in this study

Primer name	Sequence (5′-3′)	Product length (bp)	Amplification efficiency (%)
V-ATPase C-CDS-F	ATGTCTGAATACTGGGTG	1155	-
V-ATPase C-CDS-R	TTAGTTCTTGTCGAGCATGTCC		-
V-ATPase C-RFP-F	TCGTTAACACGTCAAGAGCTCATGTCT GAATACTGGGTG		-
V-ATPase C-RFP-R	TGCAGGCGCGCCGAGATCTGGTTCTTG TCGAGCATGTC		-
V-ATPase C-6P1-F	GGATCCCCGGAATTCATGTCTGAATAC TGGGTG		-
V-ATPase C-6P1-R	ATGCGGCCGCTCGAGGTTCTTGTCGAG CATGTC		-
V-ATPase C-T7-F	GATCACTAATACGACTCACTATAGGG AGAACTGTCCGATGACCTGGGTA	423	-
V-ATPase C-T7-R	GATCACTAATACGACTCACTATAGGG AGAAACATCGACTTGGGCACGAT		-
EGFP-T7-F	GATCACTAATACGACTCACTATAGGG AGACCTGAAGTTCATCTGCACCAC	586	
EGFP-T7-R	GATCACTAATACGACTCACTATAGGG AGACTCCAGCAGGACCATGTGATC		
q- V-ATPase C -F	TCGTCACCGACAAGAAGAAG	127	99

q- V-ATPase C -R	TATCTGAGCACGGACTCGAC		
EF-1α-F	GCCTGGTACCATTGTCGTCT	154	98
EF-1α-R	GTAACCACGACGCAACTCCT		
β-Actin-F	CCTGGTATTGCTGACCGTATGC	144	100
β-Actin-R	CTGTTGGAAGGTGGAGAGGGAA		

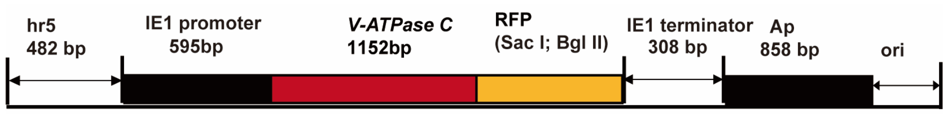

Figure. S1 The atlas of *HaV-ATPase C* -pIEx-RFP plasmid.

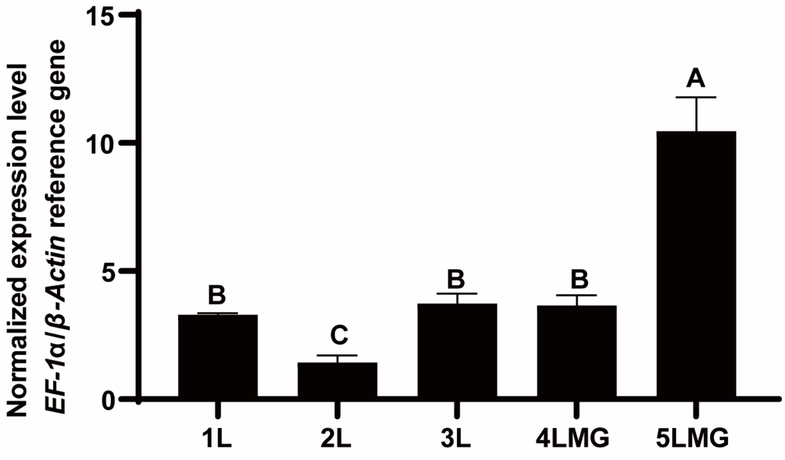

Figure. S2. The relative expression levels of the *HaVATPase C* gene in different instars of *Helicoverpa armigera.*

4LMG: MG tissues form 4 instar larvae. 5LMG: MG tissues form 5 instar larvae. The bars with different capital letters (p < 0.0001) exhibit significant differences, as determined by the least significant difference (LSD) test using DPSSOFT: DPS9.01.

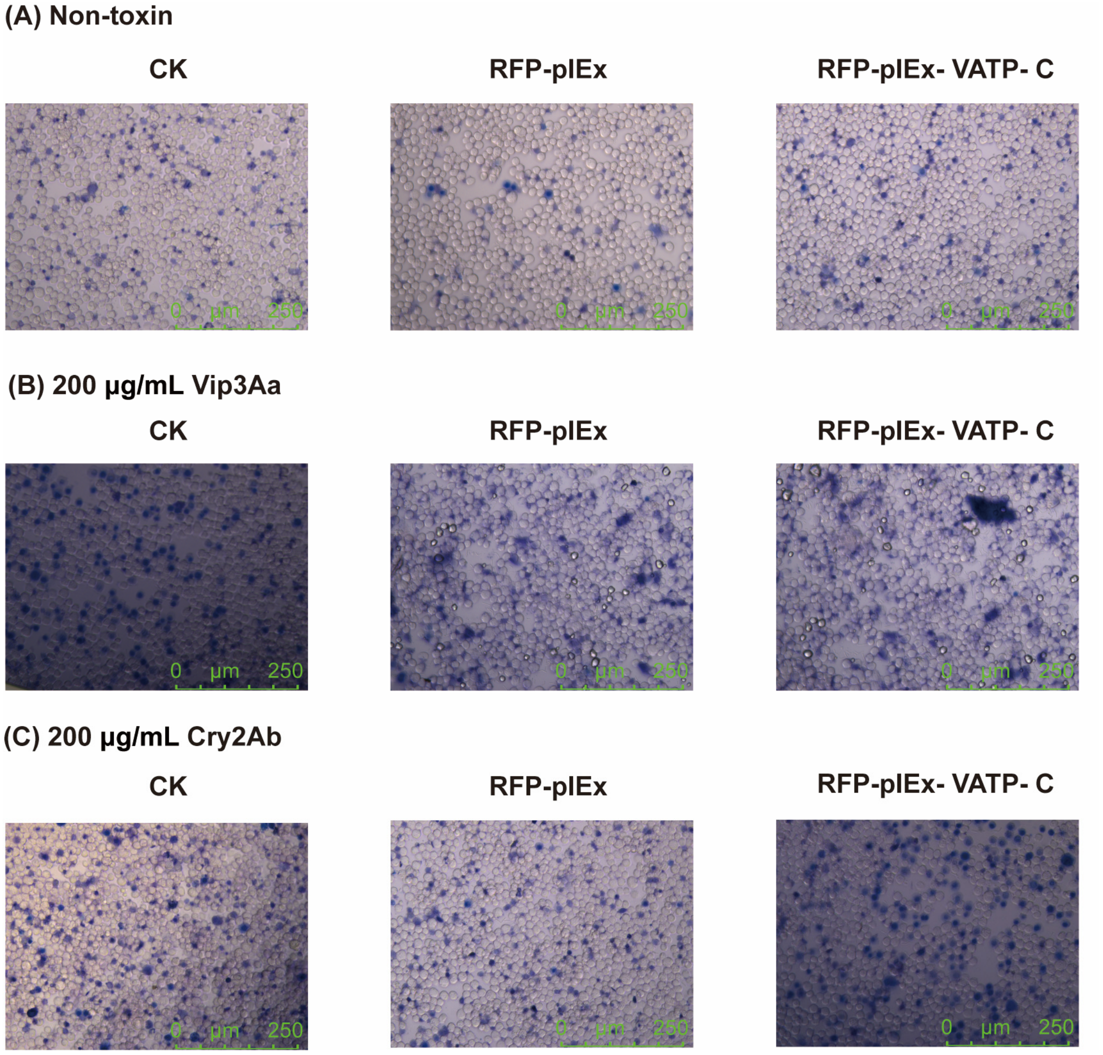

Figure. S3. Impact of expression of *HaV-ATPase C* in Sf9 cells on the cytotoxicity exerted by Cry2Ab and Vip3Aa. Photographs are representative of 400× views of the three treatments under an inverted microscope. The blue cells represent dead cells, which were stained blue by trypan blue.

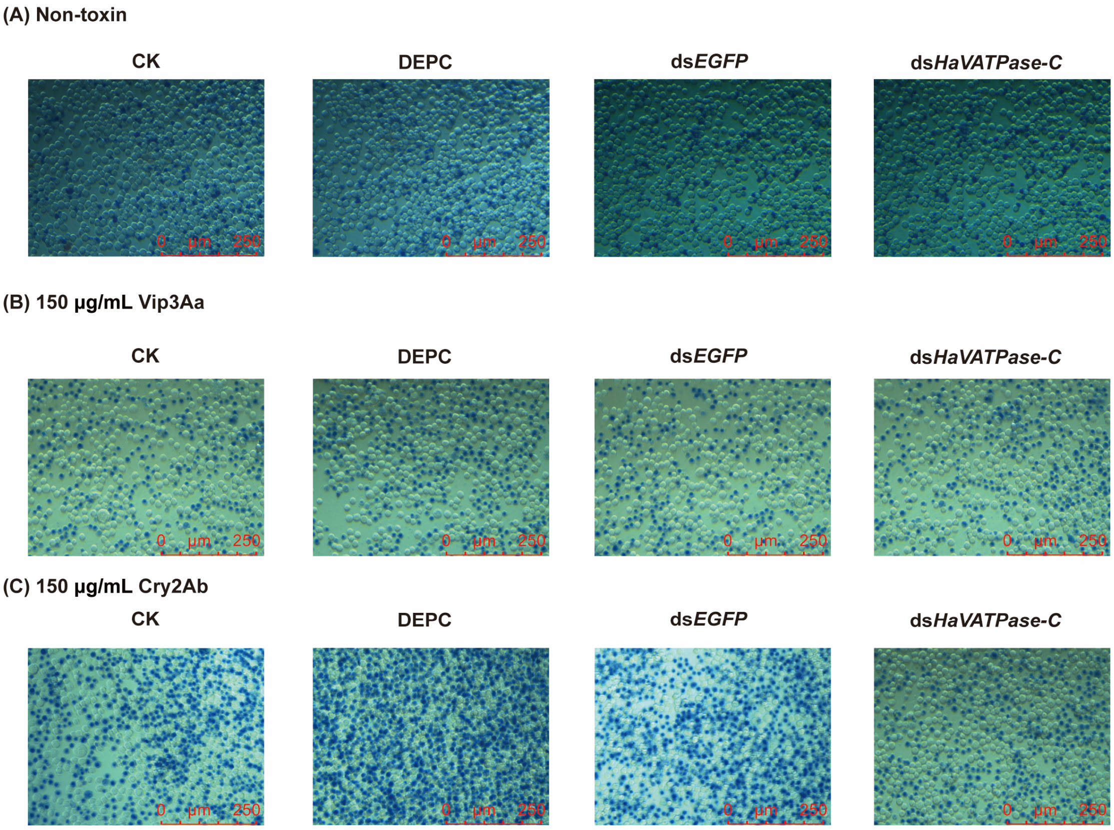

Figure. S4. Effects of *HzV-ATPase C* silencing on the toxicity of activated Cry2Ab and Vip3Aa towards MG cells. Photographs are representative of 400× views of the four treatments under an inverted microscope. The blue cells represent dead cells, which were stained blue by trypan blue.
